# Research on the synergistic modification effect and the interface mechanism of GO/SBS compound-modified asphalt based on experiments and molecular simulations

**DOI:** 10.1038/s41598-023-30593-0

**Published:** 2023-03-01

**Authors:** Qing Zeng, Yaru Liu, Qicheng Liu, Zhenghong Xu

**Affiliations:** 1 Hunan Provincial Key Laboratory of Flexible Electronic Materials Genome Engineering, School of Physics and Electronic Science, Changsha University of Science and Technology, Changsha, 410114 China; 2 School of Traffic and Transportation Engineering, Changsha University of Science and Technology, Changsha, 410114 China; 3 School of Materials Science and Engineering, Changsha University of Science and Technology, Changsha, 410114 China

**Keywords:** Engineering, Materials science

## Abstract

Although there have been reports showing the modification effect of carbon nanomaterials on asphalt, there are few studies on whether carbon nanomaterials and polymers can have synergistic modification effects on asphalt. At the same time, the complex composition of asphalt makes it difficult to determine the interface mechanism between the modifier and the asphalt. In this study, graphene oxide (GO) and styrene–butadiene–styrene block copolymer (SBS) were selected as modifiers. A combined experimental and molecular simulation research method was used to study the synergistic modification effect and the interface mechanism between the modifier and the asphalt. The results show that the modification effect of GO/SBS incorporated into asphalt is significantly superior to that of GO or SBS incorporated individually and GO/SBS has a synergistic modification effect. Although the binding strength between SBS and asphalt is weak, the GO surface (GO (0 0 1)) can simultaneously bind with SBS and asphalt, increasing the binding strength of SBS and asphalt as well as promoting the dispersion of SBS in asphalt, so that GO/SBS shows a synergistic modification effect and improves properties such as low-temperature ductility, rheology and storage stability at macroscopic level. Intercalated and exfoliated structure can be formed between GO side (GO (0 1 0)) and asphalt, which improves the anti-aging properties of the asphalt. Physical bonding is the main interface binding for GO/SBS compound-modified asphalt. GO bonds to asphalt or SBS by hydrogen bonds and there are only dispersion forces between SBS and asphalt, resulting in a higher binding strength between GO and asphalt or SBS than between SBS and asphalt.

## Introduction

Due to their excellent cohesiveness, impermeability, stable chemical structure, etc., characteristics, asphalt materials are frequently utilized and play an irreplaceable role in fields such as transportation and engineering construction^1^. However, the increasingly complex application environment makes it difficult for unmodified asphalt to meet current service requirements, and a series of problems, such as aging, cracking and pavement rutting, have gradually become prominent. Various polymers, including elastomers and thermoplastic elastomers are widely used in the field of asphalt modification^2,3^, researchers have noticed that polymers can improve the high temperature stability, resistance to permanent deformation, and fatigue life of asphalt materials^4–9^. The thermoplastic elastomer SBS is still the most common asphalt modification material^3,10^. In the past ten years, nanomaterials have also been used in asphalt modification. Incorporation of nanoparticles into polymers results in composite materials that exhibit great changes in physical and chemical properties^11,12^, and many nanomaterials (such as nano-clay, nano-zinc oxide, nano-titanium dioxide, nano-silica, etc.) can significantly improve the viscoelasticity, high-temperature stability, anti-aging performance, fatigue resistance and moisture resistance of asphalt materials^12–17^. Compared with other nanomaterials, emerging carbon nanomaterials have the advantages of low dosage characteristics with outstanding effects^18,19^ and have been shown to improve the anti-aging performance and fatigue resistance of asphalt^20,21^.

GO has unique theoretical advantages as an asphalt modifier among carbon nanomaterials. It has a 2D layered structure, which can block the oxygen from contacting the asphalt, reduce the concentration of reactants in the oxidation reaction, and improve the thermal oxidative aging performance of the asphalt^22^. A large number of oxygen-containing functional groups are distributed on GO. On one hand, the intermolecular force between the GO layers increases, which enlarges the distance between the layers, so GO aggregation is difficult^23^. On the other hand, oxygen-containing functional groups have certain reactivity, which helps GO combine with polymer molecules^24–27^. Some researchers have also modified asphalt with GO and polymers. Li et al. showed that, compared with matrix asphalt, GO has a more significant effect on improving the low-temperature crack resistance of SBS-modified asphalt^28^. Moreover, Yue et al. found that GO can improve the thermodynamic properties of asphalt binders^29^. Judging from the status of current research, although there have been studies on the effect of GO or SBS on asphalt pavement performance, there is still a lack of research on whether GO and SBS can enable synergistic modification of asphalt. Further, research on the interface bonding between GO, polymers and asphalt is even lacking.

The composition of asphalt at the microscopic level and the interaction between the different microscopic groups determines the overall properties of asphalt materials. However, popular techniques for characterizing the microstructure of asphalt often have obvious limitations, such as the lateral resolution of atomic force microscopy (AFM) is roughly 10 nm, which is too large to examine some structures like interface binding^30^. Many researchers began to use molecular simulation methods to clarify the microstructure and behavior of modified asphalt^31–33^. As the exact chemical composition of the asphalt cannot be determined, researchers have used representative molecules in molecular dynamics (MD) simulations to model the behavior of asphalt. Hansen and Lemarchand et al. proposed a four-component united-atom molecular model of asphalt and described methods for analyzing the dynamics of asphalt models by means of mean-square displacement, stress autocorrelation function and rotational relaxation^34,35^. The mesoscopic model and the 12-component model for asphalt have also been proposed by Greenfield and Li et al. which are widely applied to MD simulations of asphalt^36^, and the characteristics of the models agreed well with the elemental and saturate-aromatic-resin-asphaltene (SARA) analysis results specific to each binder^37^. Khabaz and Khare et al. investigated the glass transition, molecular mobility and ageing properties for neat and styrene-butadiene rubber (SBR) modified asphalt using the 12-component model^38^. The volume-temperature behavior of the systems exhibited a glass transition phenomenon, but the glass transition temperature of the neat and SBR-modified asphalt was not different. In addition, Khabaz and Khare successfully applied the time–temperature superposition principle to MD simulation results and construct master curves for dynamic modulus and tensile creep compliance of asphalt^39^. These models and methods proposed in the above asphalt molecular simulation investigations provide guidance for the molecular simulation of asphalt.

There are also various studies on MD simulations of nanomaterials and polymer modified asphalt. Su et al. found that ZnO/SBS microscopically mitigated the accumulation of strongly polar components and enhanced the ductility of branched chains in asphalt molecules by MD simulations^40,41^; Yu also used MD simulations to investigate the mechanism of GO's role in reducing the interaction between SBS and asphalt at the molecular level^42^. Interface bonding is the most dominant form of interaction between different microscopic groups of asphalt, however, studies of interface bonding between GO, SBS and asphalt are rarely seen.

The objective of this study was to investigate the synergistic modification of asphalt by GO/SBS and to investigate the mechanism of microscopic interface enhancement. The effects of GO/SBS on asphalt properties were characterized by experimentally investigated, and the microstructures of compound-modified asphalt were characterized by X-ray diffraction (XRD), Fourier infrared spectroscopy (FTIR) and fluorescent micrograph. Finally, the interface structure models of compound-modified asphalt were constructed, and the microscopic interface enhancement mechanism was revealed by MD simulation and first-principles calculation.

## Experimental and simulation details

Please refer to supplementary materials for raw materials, equipment models and test methods used in the experiments.

### Preparation of samples

#### Preparation of SBS and GO modified asphalt

The SBS-modified asphalt was prepared using a high shear mixer. First, matrix asphalt was heated to a completely molten state at 150 °C. Then, different mass fractions of SBS were added to the asphalt, and a high-speed homogenizer was used for shearing and stirring at 6000 rpm for 30 min. Finally, the asphalt was cooled to room temperature to obtain SBS-modified asphalt. The unmodified asphalt is referred to as 70A, and the modified asphalt with a content of 1–13 wt% SBS is referred to as 70A + (1–13%)SBS.

GO-modified asphalt was prepared similarly to SBS-modified asphalt. First, matrix asphalt was heated to a completely molten state at 150 °C. Then, different mass fractions of GO were added to the asphalt, and a high-speed homogenizer was used for shearing and stirring at 6000 rpm for 30 min. Finally, the asphalt was cooled to room temperature to obtain GO-modified asphalt. The modified asphalt with a content of 0.05–0.5 wt% GO is referred to as 70A + (0.05–0.5%)GO.

#### Preparation of GO/SBS compound-modified asphalt

The GO/SBS compound-modified asphalt was prepared using a high shear mixer at 150 °C and a shearing speed of 6000 rpm. First, matrix asphalt was heated to a completely molten state at 150 °C, and different mass fractions of SBS were incorporated and stirred until the SBS and asphalt were evenly mixed. Then, different mass fractions of GO were added, and a high-speed homogenizer was used for shearing and stirring at 6000 rpm for 40 min. Finally, the asphalt was cooled to room temperature to obtain GO/SBS compound-modified asphalt. The compound-modified asphalt with a content of 1–13 wt% SBS and 0.05–0.5 wt% GO is referred to as 70A + (1–13%)SBS + (0.05–0.5%)GO.

### Molecular simulation models and methods

#### Construction of models

Asphalt is an extremely complex hydrocarbon. Due to the complex structure, it is not enough to identify and quantify the specific chemical parts of asphalt in experiments^43^. Nevertheless, previous studies have shown that using the average molecular structures based on experimental data to represent actual asphalt is a feasible and effective method for studying the physical and chemical properties of asphalt^44^. According to the asphalt four-component classification method, some asphalt researchers have constructed molecular models of saturate, aromatic, resin, and asphaltene, which were used to perform MD research on asphalt^45–47^, thus confirming the feasibility of this molecular simulation method to study asphalt materials. In this study, molecular models of saturate, aromatic, resin, and asphaltene were constructed based on references^45–47^, where saturate components were further divided into linear saturate and naphthenic saturate^48,49^. The molecular structure, molecular formula, number of molecules and content of each component in the asphalt model are shown in Table 1.

**Table 1:** Models of SBS, GO and each component of asphalt. (White, gray and red on GO represent hydrogen, carbon and oxygen atoms, respectively).

Component		Structure	Molecular formula	Number of molecules	Content (wt%)
Saturate	Linear saturate		C_22_H_46_	4	10
	Naphthenic saturate		C_24_H_46_	2	5.4
Aromatic			C_48_H_52_O_2_	7	37.2
Resin			C_55_H_71_NO_2_S	5	32.6
Asphaltene			C_129_H_145_N_3_O_2_S_2_	1	14.8
SBS			C_140_H_180_	6	–
GO	GO(0 0 1)		–	–	–
	GO(0 1 0)		–	–	–

A linear SBS molecular model was constructed based on the raw SBS materials used in the experiment (where the styrene : butadiene ratio is 8:19 which is approximately equal to 30:70). To study the GO interface in modified asphalt as comprehensively as possible, this paper divided GO into two surface structures: the GO surface (GO (0 0 1)) and the GO side (GO (0 1 0)), details of GO (0 0 1) and GO (0 1 0) are shown in Fig. S1. While the surface of GO is mainly distributed with hydroxyl and epoxy groups, the edge of GO is mainly distributed with carboxyl groups^26^. This study constructed GO (0 0 1) and GO (0 1 0) models based on these typical GO chemical characteristics. The SBS and GO structures are shown in Table 1.

**Figure 1:**
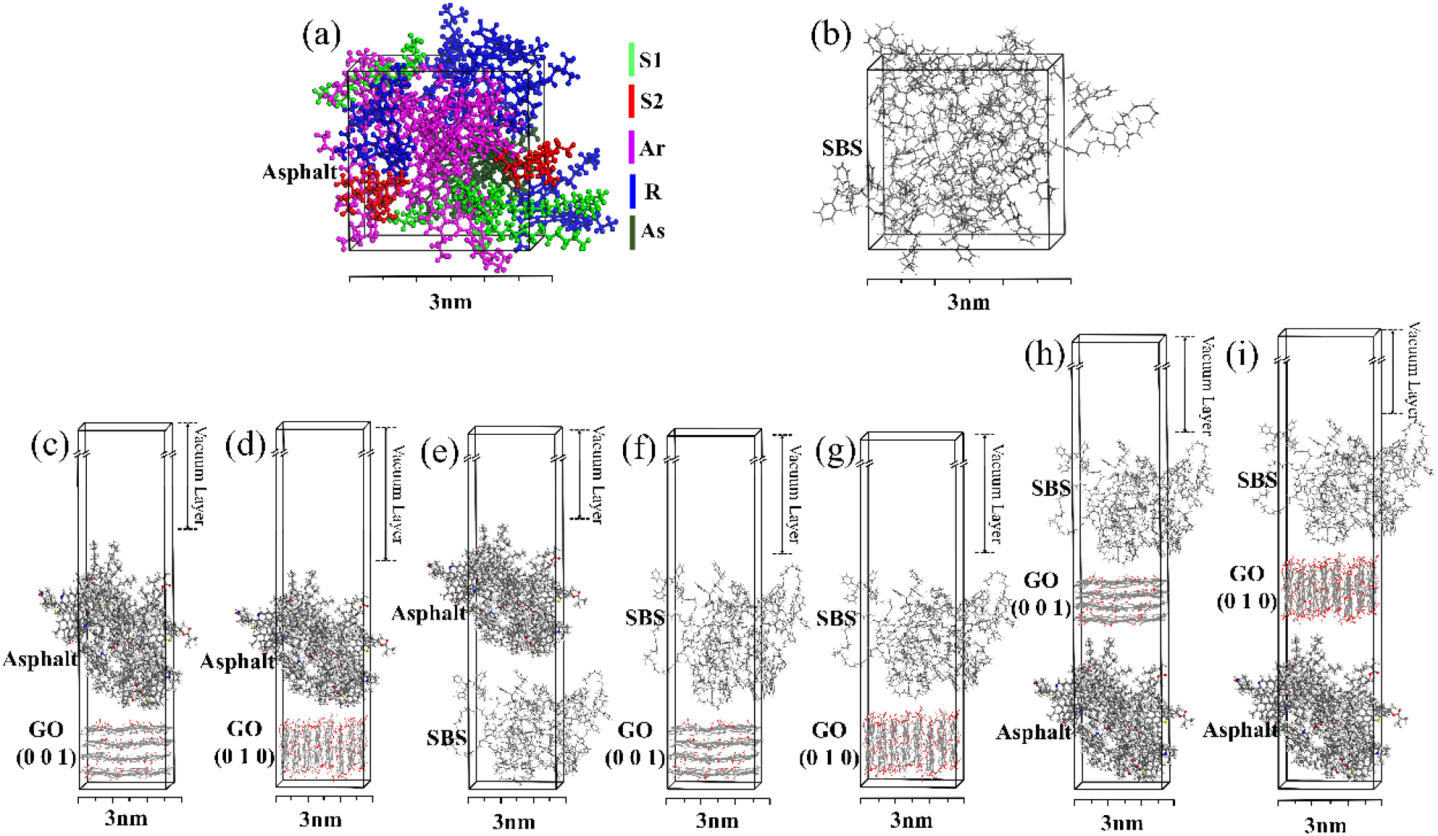
(**a**) Asphalt model, (**b**) SBS model, (**c**–**g**) interface models and (**h**,**i**) compound interface models.

The asphalt cell model was constructed based on the content of each asphalt component in Table 1, as shown in Fig. 1(a). Figure 1(b) shows the constructed SBS cell model.

#### Calculation methods and parameters

The asphalt and SBS cell models (Fig. 1a,b) were subjected to geometry optimization, annealing, and dynamic relaxation in sequence to ensure that reasonable structures were obtained. The optimized models were used to construct GO (0 0 1)/asphalt, GO (0 1 0)/asphalt, SBS/asphalt, GO (0 0 1)/SBS and GO (0 1 0)/SBS interface models, as shown in Fig. 1(c–g), and asphalt/GO (0 0 1)/SBS, asphalt/GO (0 1 0)/SBS compound interface models, as shown in Fig. 1(h,i). To establish the interface structure, all models were given a 10 nm vacuum layer along the vertical direction. Considering that modified asphalt was prepared at a temperature of 150  °C, the interface structures were simulated by MD simulations at 100 °C, 125 °C and 150 °C to analyze the interface microstructures and mechanisms between asphalt and modifier.

MD simulations were performed using the Forceite package of Materials Studio. In this study, the components included in the simulation system are SBS, GO and asphalt, where SBS and asphalt are organic macromolecules and GO is an inorganic non-metal consisting of carbon and oxygen. The Dreiding force field provides excellent coverage of organic macromolecules and main group inorganic materials which can provide an accurate description of the system for this study and has been used to simulate asphalt in other studies^33,50^. Therefore, Dreiding force field and Charge Equilibration (QEq) were used, the atom-based summation with a cutoff distance of 12.5 Å and the Ewald method with a cutoff distance of 12.5 Å were adopted to calculate van der Waals and electrostatic interactions, respectively. The temperature and pressure of the systems were controlled by using Nosé-Hoover thermostat and barostat. The time step was chosen to be 0.001 ps for all the stages. In annealing, the cycle temperature range was 27–227 °C, and the total number of annealing cycles to be performed was specified as 5. All systems used for simulations were subjected to 1200 ps MD relaxation under the Isothermal-isobaric ensemble (NPT ensemble) before analysis. The changes in the properties of the model during the annealing and MD simulations are shown in Figs. S2, S3. The system properties of all interface models were in equilibrium and in a steady state within the range of 800 ps to 1200 ps (last 400 ps) of MD relaxation. Therefore, the structures in the range of 800 ps to 1200 ps in Figs. S2c,d and S3 were selected for analysis.

After the MD simulation, stable structures were extracted. The stable interface models were geometry optimized based on first-principles calculations, and then the atomic charge distribution, charge density and charge density difference of the interface were obtained to analyze the physical and chemical properties.

First-principles calculations were performed using the Dmol3 package of Materials Studio. The generalized gradient approximation (GGA) with the Perdew-Burke-Ernzerhof (PBE) exchange–correlation function in density functional theory (DFT) and double numerical plus (DNP) polarization atomic orbitals of the basis set were employed, with the energy tolerance accuracy set at 2.723 × 10^−4^ eV. To evaluate the intermolecular force, dispersion correction (DFT-D) was used to correct the dispersion force. For more details, please see the DFT-D section in supplementary materials.

Each interface model in Fig. 1(c–g) is presented with components of *A* and *B*. The binding energies *E*_*A/B*_ between *A* and *B* were calculated using the following formula:

1 $${E}_{A/B}={E}_{A-B}-{(E}_{A}+{E}_{B})$$

where the parameter *E*_*A-B*_ represents the total energy of the system after *A* and *B* are bonded and *E*_*A*_ and *E*_*B*_ are the total energy of *A* and *B* individually, respectively.

Each compound interface model in Fig. 1(h,i) is presented with components of *A B* and* C*. The binding energies *E*_*A-B/C*_ of the compound interface in Fig. 1(h,i) are shown in the following formula:

2 $${E}_{A-B/C}={E}_{A-B-C}-{(E}_{A-B}+{E}_{C})$$

where the parameter *E*_*A-B-C*_ represents the total energy of the system after the bonding of *A*, *B* and *C*, *E*_*A-B*_ represents the total energy of the system after *A* and *B* are bonded, and *E*_*C*_ is the total energy of* C* individually. In this paper, *A, B,* and* C* each either represent asphalt, GO (0 0 1), GO (0 1 0) or SBS.

## Results and discussion

### Performance analysis

#### Physical properties

The effects of GO and SBS content on the physical properties of the modified asphalt are shown in Table 2. The softening point of SBS-modified asphalt increases with increasing SBS contents, it increases slowly when the SBS contents are less than 3% and more than 9%. However, the softening point shows a rapid increase when the SBS contents are between 3 and 9 wt%. In contrast, the penetration of the SBS-modified asphalt decreases with increasing SBS contents, especially in the range of 5 wt% to 9 wt%. The low-temperature ductility of SBS-modified asphalt first increases and then decreases with increasing SBS contents, and it increases significantly when the SBS contents are less than 5 wt% but shows a rapid decrease when the SBS contents are more than 5 wt%.

**Table 2:** Effects of GO and SBS on the physical properties of asphalt. (Three equal samples are tested for different properties; the data in the table are average values and numbers in the parentheses show the standard deviation).

Asphalt	Softening point (°C)	Penetration at 25 °C (mm)	Ductility at 5 °C (mm)
70A	48.6 (0.047)	7.102 (0.134)	85 (0.471)
70A + 1%SBS	50.0 (0.163)	5.849 (0.097)	72 (0.816)
70A + 3%SBS	53.4 (0.082)	5.650 (0.077)	98 (0.816)
70A + 5%SBS	61.3 (0.245)	5.025 (0.079)	179 (1.633)
70A + 7%SBS	73.0 (0.327)	3.680 (0.063)	48 (0.816)
70A + 9%SBS	86.2 (0.498)	2.707 (0.058)	19 (0.471)
70A + 11%SBS	91.6 (0.408)	2.350 (0.050)	13 (0.471)
70A + 13%SBS	95.3 (0.368)	2.013 (0.044)	14 (0.816)
70A + 0.05%GO	49.2 (0.047)	6.898 (0.141)	61 (1.633)
70A + 0.2%GO	49.6 (0.082)	6.951 (0.153)	62 (0.471)
70A + 0.5%GO	49.8 (0.125)	6.321 (0.113)	59 (0.471)
70A + 5%SBS + 0.2%GO	71.6 (0.163)	5.036 (0.083)	241 (3.682)
70A + 5%SBS + 0.5%GO	72.6 (0.205)	5.012 (0.062)	243 (3.266)
70A + 9%SBS + 0.2%GO	87.5 (0.125)	2.572 (0.035)	24 (0.816)
70A + 9%SBS + 0.5%GO	88.2 (0.205)	2.498 (0.051)	14 (0.816)

GO slightly increases the softening point, decreases the penetration and low-temperature ductility of the asphalt. Compared to the incorporation of SBS individually, the incorporation of GO individually has a weaker influence on asphalt properties. However, the softening point and low-temperature ductility of 70A + 5%SBS + (0.2–0.5%)GO show a significant increase compared with those of 70A + 5%SBS. The softening point of 70A + 5%SBS and 70A + 0.2%GO are 61.3 °C and 49.6 °C respectively, which are 12.7 °C and 1 °C higher than that of 70A, while, the softening point of 70A + 5%SBS + 0.2%GO is 23 °C higher than that of 70 A, at 71.6 °C. In other words, the increase in softening point or low-temperature ductility of the modified asphalt when GO and SBS are incorporated together is greater than the sum of that when GO and SBS are incorporated individually. GO/SBS show a synergistic modification effect on asphalt when the SBS content on 5wt%; and the performance of the compound-modified asphalt has been significantly improved.

The performance of 70A + 9%SBS + (0.2–0.5%)GO does not change much compared to that of 70A + 9%SBS, and the GO/SBS synergistic modification effect is most evident at 5wt% SBS content. Meanwhile, the physical properties of 70A + 5%SBS + 0.5%GO are similar to those of 70A + 5%SBS + 0.2%GO, so the optimum contents of GO and SBS are 0.2wt% and 5wt%, respectively.

#### Aging properties

Table 3 shows the asphalt physical properties measured before and after RTFO aging. Compared with 70A, the 70A + 0.2%GO softening point increment is reduced from 3.8 °C to 1.9 °C, the residual penetration is increased from 65.87% to 72.51%, and the residual ductility at 5 °C is increased from 27.17% to 43.32% after aging, thus GO improves the thermal oxidative aging resistance of the asphalt matrix. However, the softening point and ductility at 5 °C for 70A + 5%SBS + 0.2%GO show more significant changes with ageing than those for 70A + 5%SBS. Therefore, GO is unsatisfactory for improving the thermal oxygen ageing resistance of SBS-modified asphalt.

**Table 3:** RTFO aging test results. (Three equal samples are tested for different properties; the data in the table are average values and numbers in the parentheses show the standard deviation).

Asphalt	Softening point (°C)		Penetration at 25 °C (mm)		Ductility at 5 °C (mm)	
	Unaged	aged	Unaged	aged	Unaged	aged
70A	48.6 (0.047)	52.4 (0.125)	7.102 (0.134)	4.678 (0.060)	85 (0.471)	23 (0.471)
70A + 0.2%GO	49.6 (0.082)	51.5 (0.125)	6.951 (0.153)	5.04 (0.056)	62 (0.471)	27 (0.816)
70A + 5%SBS	61.3 (0.245)	58.6 (0.163)	5.025 (0.079)	4.001 (0.062)	179 (1.633)	13 (0.471)
70A + 5%SBS + 0.2%GO	71.6 (0.163)	58.1 (0.047)	5.036 (0.083)	4.042 (0.054)	241 (3.682)	35 (1.247)

#### Viscosity properties

**Figure 2:**
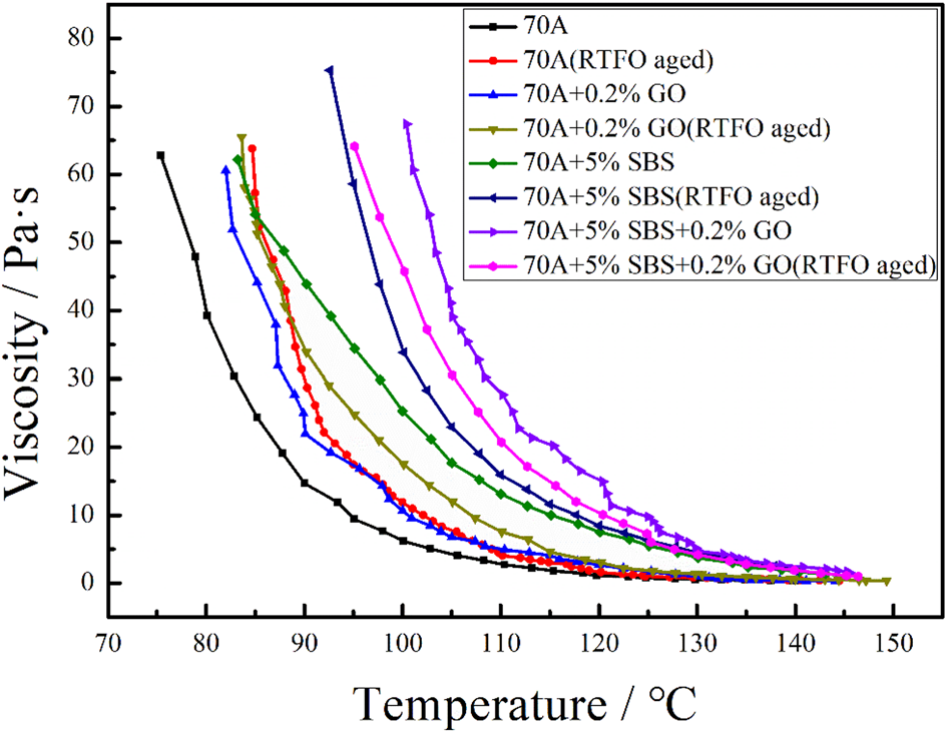
Viscosity-temperature curves of modified asphalt and aged modified asphalt.

Figure 2 shows the viscosity-temperature curves of modified asphalt and aged modified asphalt. The viscosity of 70A + 5%SBS + 0.2%GO shows an evident increase compared to the viscosities of 70A + 0.2%GO and 70A + 5%SBS. In addition, the increase in viscosity of the 70A + 5%SBS + 0.2%GO is greater than the sum of that in 70A + 0.2%GO and 70A + 5%SBS compared to 70A. For example, the viscosities of 70A, 70A + 5%SBS, 70A + 0.2%GO and 70A + 5%SBS + 0.2%GO at 110 °C are 2.691 Pa s, 13.108 Pa s, 4.902 Pa s and 27.688 Pa s, respectively. The viscosity of 70A + 5%SBS and 70A + 0.2%GO are 10.417 Pa s and 2.211 Pa s higher than that of 70A respectively, and the sum of the viscosity increments value is less than that of 70A + 5%SBS + 0.2%GO, which is 24.997 Pa s. This indicates that GO/SBS can have a synergistic effect on asphalt. For the viscosity of aged modified asphalt, the viscosities of 70A, 70A + 0.2%GO and 70A + 5%SBS all increase after aging. It is worth noting that the viscosity of aged 70A + 5%SBS + 0.2%GO is lower than that of unaged 70A + 5%SBS + 0.2%GO, and the possible causes of this phenomenon are explained by MD simulations of the interface, as detailed in "Concentration distribution of interface".

#### Storage stability properties

Table 4 shows the results of the segregation test of modified asphalt. The softening point difference between the top section and bottom section of 70A + 0.2%GO is 0.7 °C, which is slightly higher than the 0.2 °C of 70A, but the softening point difference of 70A + 5%SBS reached 2.4 °C, much higher than that of 70A at 0.2 °C. Although the application of SBS as the modifier leads to poorer storage stability of the modified asphalt, SBS has significant potential to improve the performance of the asphalt, therefore, the storage stability of SBS-modified asphalt needs to be improved as much as possible while maintaining the modification effect. The softening point difference between the top section and bottom section of 70A + 5%SBS + 0.2%GO is clearly less than that of 70A + 5%SBS, which means that GO prevents the segregation of SBS in the asphalt. GO/SBS as the modifier contributes more to the overall performance of the modified asphalt. In the "Binding energy of interface", the reasons for GO improving the storage stability of SBS-modified asphalt are analyzed by means of interface binding energy.

**Table 4:** Separation test results of modified asphalt. (Three equal samples are tested for softening point; the data in the table are average values and numbers in the parentheses show the standard deviation).

Parameter	70A	70A + 0.2%GO	70A + 5%SBS	70A + 5%SBS + 0.2%GO
Top section (°C)	49.0 (0.082)	49.2 (0.047)	62.7 (0.245)	72.5 (0.125)
Bottom section (°C)	48.8 (0.082)	49.9 (0.163)	60.3 (0.125)	71.0 (0.125)
Softening point difference (°C)	0.2	0.7	2.4	1.5

### Microstructure analysis

#### XRD analysis

**Figure 3:**
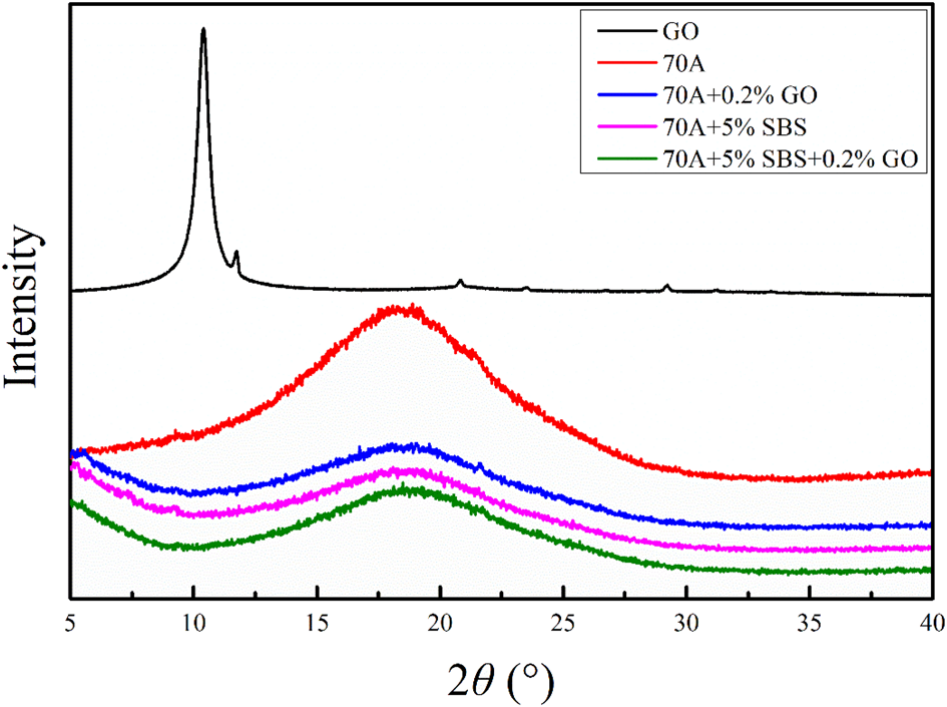
XRD images of the GO, matrix asphalt and modified asphalt.

Figure 3 shows the XRD images of the GO, matrix asphalt and modified asphalt. The XRD images underwent longitudinal translation to improve their readability. The diffraction peak of the GO (0 0 2) crystal plane corresponds to 2*θ* = 10.405°, which shows that the interlayer distance of GO is approximately 0.851 nm. The XRD curves of 70A + 0.2%GO and 70A + 5%SBS + 0.2%GO did not show the characteristic GO peak near 2*θ* = 10.405°. This may be caused by either destruction of the GO lamellae structure and a decrease in the degree of crystallinity or low GO content that cannot be characterized. In addition, the characteristic peaks (γ peaks) of 70A, 70A + 0.2%GO, 70A + 5%SBS and 70A + 5%SBS + 0.2%GO at 2*θ* are located near 18.8826°, 19.0206°, 18.094° and 18.4883°, respectively. These peaks may mainly be caused by the structure of the saturated part or the alkyl chain^51,52^. The γ peak intensity of 70A is significantly stronger than those of 70A + 0.2%GO, 70A + 5%SBS and 70A + 5%SBS + 0.2%GO, which indicates that the saturated chain crystallinity of modified asphalt decreases after GO or SBS is incorporated. The possible reasons for the reduced crystallinity of the saturated chains have been explained by first principles calculations of interface, as detailed in "First-principles calculation of interface".

#### FTIR analysis

**Figure 4:**
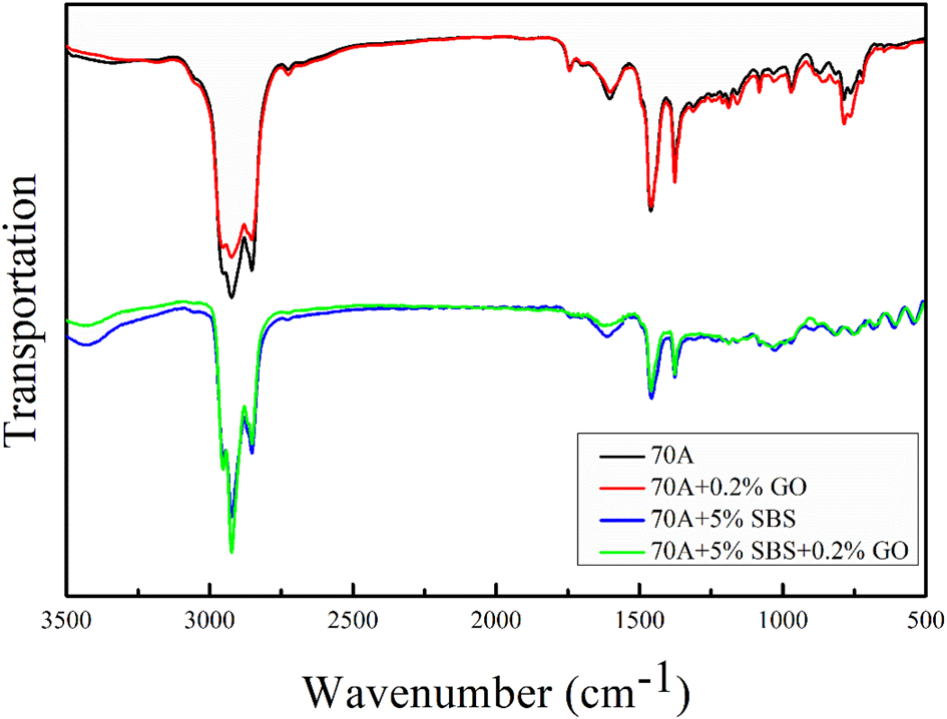
FTIR images of matrix asphalt and modified asphalt.

Figure 4 shows the FTIR images of 70A, 70A + 0.2%GO, 70A + 5%SBS and 70A + 5%SBS + 0.2%GO. The FTIR images of 70A + 0.2%GO and 70A have the same absorption bands, and the main absorption bands of 70A + 5%SBS and 70A + 5%SBS + 0.2%GO are also equivalent. Although the content of GO in the asphalt is very low and some functional groups with low content in the modified asphalt may not be visible, it can be determined that GO has no significant effect on the vast majority of the SBS and asphalt functional groups by FTIR, and physical modification may dominate. In fact, the results of the first-principles calculations (in "First-principles calculation of interface") show that all interfaces between GO, SBS and asphalt are physically bound.

#### Fluorescent micrograph analysis

**Figure 5:**
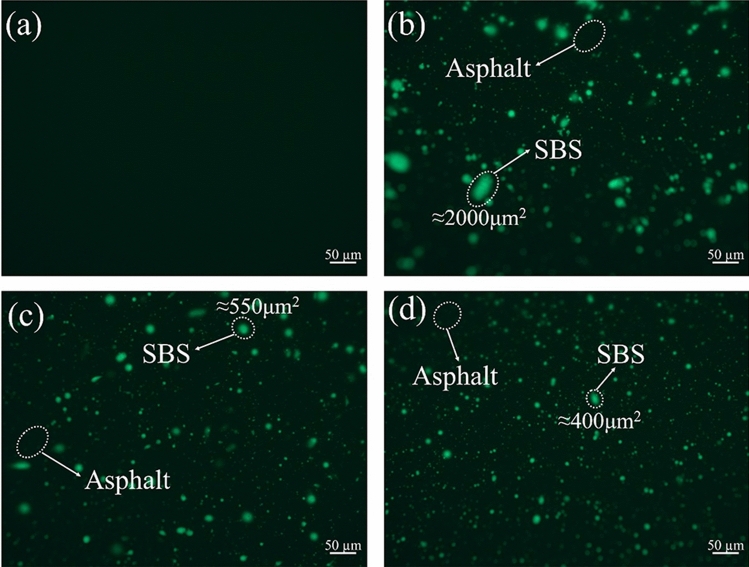
Fluorescence micrographs of (**a**) 70A + 0.2%GO, (**b**) 70A + 5%SBS, (**c**) 70A + 5%SBS + 0.2%GO and (**d**) 70A + 5%SBS + 0.5%GO.

Fluorescent micrographs of 70A + 0.2%GO, 70A + 5%SBS, 70A + 5%SBS + 0.2%GO and 70A + 5%SBS + 0.5%GO are shown in Fig. 5. The SBS modifier is shown as a highlighted area in the micrograph. 70A + 0.2%GO does not show fluorescence to serve as a comparison, as shown in Fig. 5(a). The micrograph of 70A + 5%SBS shows large SBS particle sizes with the maximum size of approximately 2000 μm^2^, as shown in Fig. 5(b), indicating significant agglomeration of the SBS modifier in the asphalt. The maximum size of SBS particles in 70A + 5%SBS + 0.2%GO and 70A + 5%SBS + 0.5%GO micrographs are approximately 550 μm^2^ and 400 μm^2^ respectively, as shown in Fig. 5(c,d). The size of the SBS particles decreases significantly with the incorporation of GO, indicating that GO facilitates the dispersion of SBS in asphalt. The microstructures of 70A + 5%SBS, 70A + 5%SBS + 0.2%GO and 70A + 5%SBS + 0.5%GO are consistent with the results of the physical properties tests. The synergistic modification of GO/SBS is reflected in GO promoting the dispersion of SBS and giving a more uniform distribution of SBS in the asphalt, resulting in a further increase in softening point and low-temperature ductility for 70A + 5%SBS + 0.2%GO and 70A + 5%SBS + 0.5%GO compared to 70A + 5%SBS.

### Molecular simulation analysis

#### Binding energy of interface

**Figure 6:**
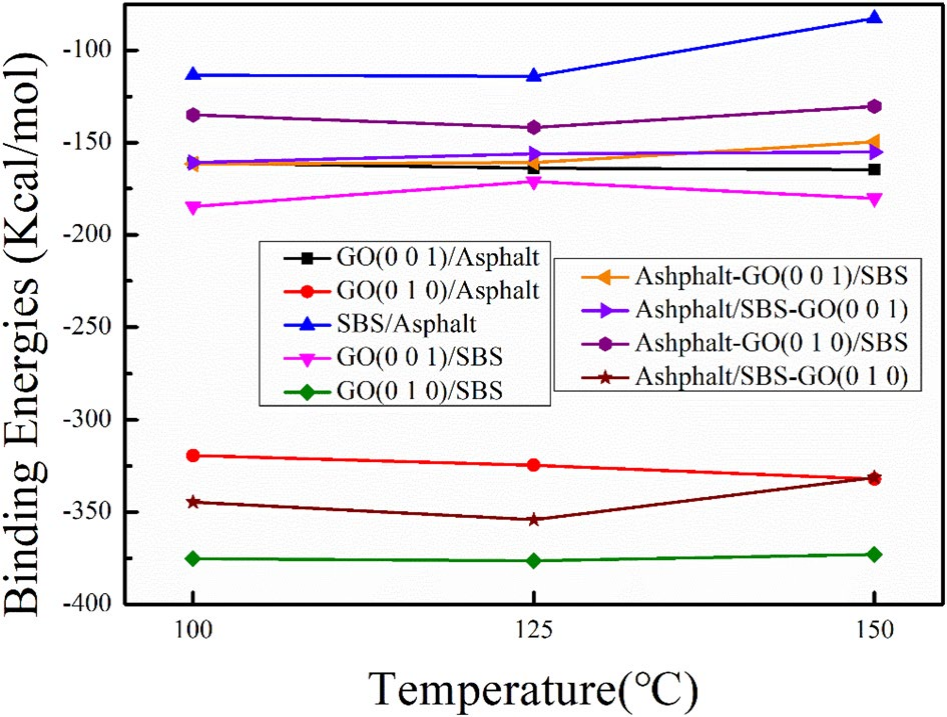
Binding energies between each interface of the GO/SBS compound-modified asphalt.

Figure 6 shows the binding energies of each interface that may exist in the GO/SBS compound-modified asphalt at temperatures of 100 °C, 125 °C and 150 °C. Detailed data for binding energies are shown in Table S2. For the simple interfaces (Fig. 1c–g), the results show that all interface binding energies are less than zero, which indicates that any pairwise binding of GO, SBS and asphalt involves a process of releasing energy, allowing the binding to proceed spontaneously. The binding energies of GO (0 1 0)/asphalt and GO (0 0 1)/asphalt are both smaller than that of SBS/asphalt, which means that the bonding strength between GO and asphalt is higher than that between SBS and asphalt, and the highest bonding strength is between GO (0 1 0) and asphalt. At the same time, the binding energies of GO (0 1 0)/SBS and GO (0 0 1)/SBS are also smaller than that of SBS/asphalt, indicating that the bonding strength between GO and SBS is still stronger than that between asphalt and SBS. Therefore, the interface binding strength between SBS and asphalt is the weakest, followed by that between GO (0 0 1) and asphalt or SBS, and the strongest interface bonding strength appears between GO (0 1 0) and asphalt or SBS. For the compound interfaces (Fig. 1h,i), the binding energies of asphalt-GO (0 0 1)/SBS, asphalt/SBS-GO (0 0 1), asphalt-GO (0 1 0)/SBS and asphalt/GO (0 1 0)-SBS are all less than the binding energy of asphalt/SBS, which indicates that the bonding strength between asphalt and SBS is enhanced after GO is incorporated. Moreover, the binding energy of asphalt/SBS increases with increasing temperature (100 °C: − 113.430 kcal/mol, 150 °C: − 82.72 kcal/mol), while the binding energies of asphalt-GO (0 0 1)/SBS, asphalt/SBS-GO (0 0 1) and asphalt-GO (0 1 0)/SBS exhibit only small changes with increasing temperature. This means that the influence of temperature changes on the binding strength between asphalt and SBS is reduced with the incorporation of GO.

The experimental results in "Performance analysis" show that GO alone cannot improve asphalt performance to a large extent and that SBS is still the key to improving asphalt performance. However, the results in Fig. 6 show that the interface binding between SBS and asphalt is weak, and the weak SBS/asphalt interface leads to easy separation between SBS and asphalt (corresponding to Fig. 5b), SBS tends to agglomerate in the asphalt and cannot fully develop the modification effect of SBS. With the incorporation of GO, the interface bonding strength between SBS and asphalt is effectively enhanced. This stronger interface bonding leads to difficult separation between SBS and asphalt and promotes a more uniform distribution of SBS in the asphalt (corresponding to Fig. 5c,d), which is also responsible for the synergistic modification effect shown by GO/SBS and resulting in further improvements of properties such as softening point, low-temperature ductility, viscosity and especially storage stability of 70A + 5%SBS + 0.2%GO compared to 70A + 5%SBS in macroscopic experiments.

#### Concentration distribution of interface

**Figure 7:**
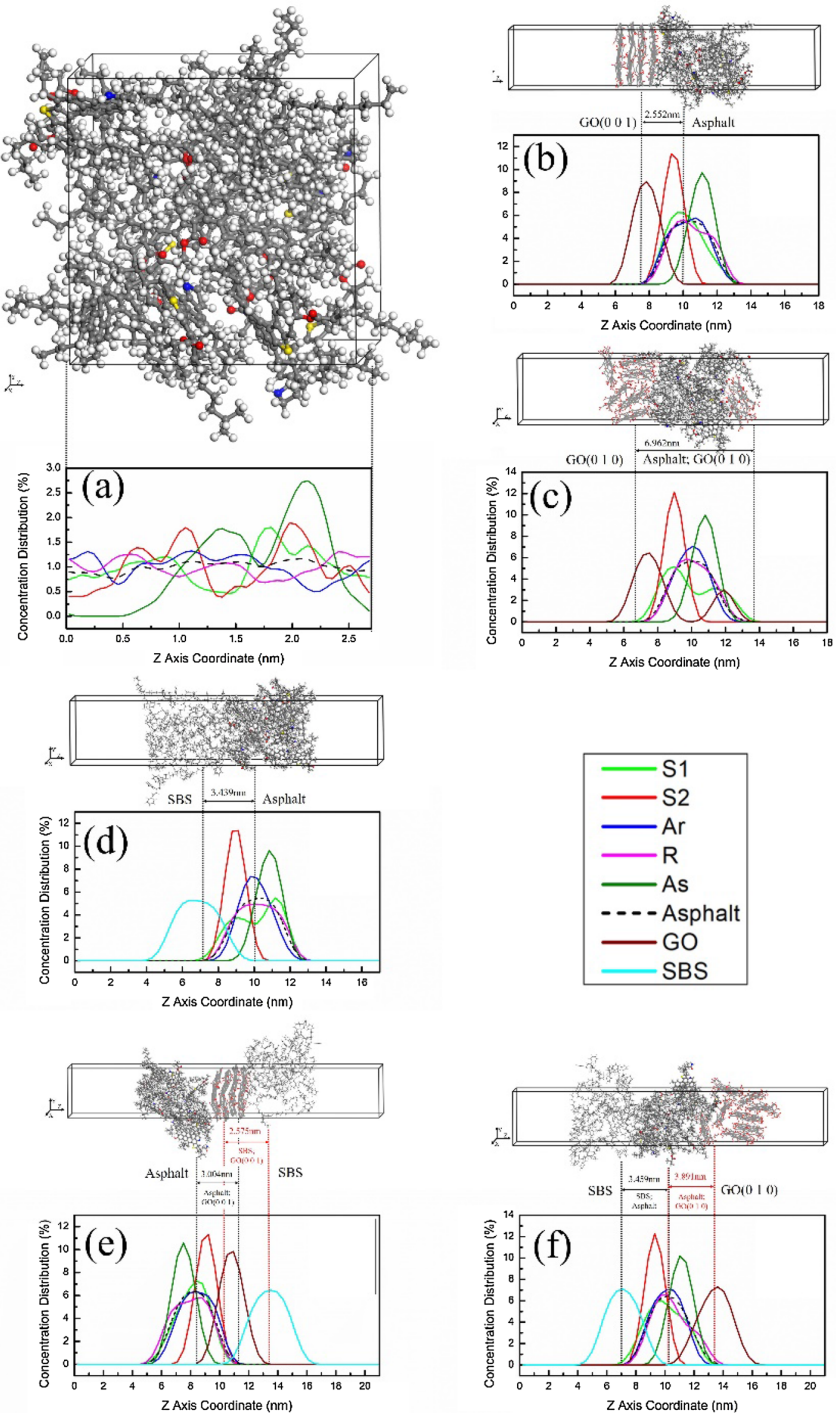
Interface molecular concentration distribution of (**a**) asphalt, (**b**) GO (0 0 1)/asphalt, (**c**) GO (0 1 0)/asphalt, (**d**) SBS/asphalt, (**e**) asphalt/GO (0 0 1)/SBS and (**f**) asphalt/GO (0 1 0)/SBS.

The lowest binding energy of interface means the most stable interface binding. As can be seen from the data in Table S2, the asphalt interface of GO (0 0 1)/Asphalt, GO (0 1 0)/Asphalt, SBS/Asphalt, Asphalt-GO (0 0 1)/SBS (or Asphalt/SBS-GO (0 0 1)) and Asphalt-GO(0 1 0)/SBS (or Asphalt/SBS-GO(0 1 0) have the lowest interface binding energy at 150 °C, 150 °C, 125 °C, 100 °C and 125 °C, respectively. The interfaces between GO (0 0 1), GO (0 1 0), SBS and asphalt with the lowest binding energy were selected to analyze the molecular concentration distribution and clarify the interactions between asphalt components and modifiers. To improve readability, the interface model is translated along the Z axis, as shown in Fig. 7(b–f). S1, S2, Ar, R and As represent linear saturate, naphthenic saturate, aromatic, resin and asphaltene components of asphalt, respectively.

Figure 7(a) shows the molecular concentration distribution of the asphalt model along the normal direction of the binding surface (Z-axis direction). The saturate, aromatic and resin are roughly in a disordered state, indicating that the components of the asphalt do not have a definite distribution pattern before binding with GO or SBS. Figure 7(b) shows the molecular concentration distribution at the GO (0 0 1)/asphalt interface. GO (0 0 1) and asphaltene (As) are concentrated at 7.839 nm and 11.121 nm, respectively, and saturate (S1, S2), aromatic (Ar) and resin (R) molecules are sequentially distributed in the range of 7.839–11.121 nm. The concentration distribution shows that the resin and aromatic are gathered around asphaltene and dispersed in the saturate. GO (0 0 1) is in contact with saturate, aromatic and resin molecules surrounding the asphaltene, where the saturate is the main binding component. The cross-concentration between the GO (0 0 1) and asphalt is 2.552 nm.

Figure 7(c) displays the molecular concentration distribution at the GO (0 1 0)/asphalt interface. GO (0 1 0) is distributed mainly at approximately 7.511 nm and 11.908 nm. Although the GO (0 1 0) distributed at approximately 7.511 nm is in contact with saturate, aromatic and resin surrounding the asphaltene, it remains mainly binding to saturate. Combination with the structure model shows that some linear alkanes enter the GO sheet at 7.511 nm to form an intercalated structure. The GO at 7.511 nm is still in an aggregated state, but its layered structure is damaged to a certain extent. The GO at 11.908 nm is exfoliated from the sheet to form an exfoliated structure. The exfoliated GO enters the asphalt micelles and gathers linear saturate molecules (S1). Due to the exfoliation of GO, the cross-concentration between GO (0 1 0) and asphalt is 6.962 nm.

The intercalated and exfoliated structure formed between GO and asphalt, coupled with the high aspect ratio of GO, greatly hinders the movement of asphalt molecular chains. At the same time, these two microstructures can inhibit the volatilization and oxidation of the saturated components during the thermal oxygen aging process. Therefore, 70A + 0.2%GO has a higher viscosity and better anti-aging performance than 70A.

Figure 7(d) shows the molecular concentration distribution at the SBS/asphalt interface. SBS and asphaltene are concentrated at 6.707 nm and 10.834 nm, respectively, and saturate, aromatic and resin molecules are sequentially distributed in the range of 6.707–10.834 nm. Resin and aromatic are gathered around asphaltene and dispersed in the saturate, and the SBS is in contact with the saturate, aromatic and resin molecules surrounding the asphaltenes. The cross-concentration between SBS and asphalt is 3.429 nm. The concentration peaks of saturate components appear near 9.114 nm, indicating that SBS tends to dissolve in the light components of asphalt and hardly contacts the asphaltene molecules. The SBS and the asphaltene are independent of each other, which is consistent with the current research results on the colloidal structure of polymer-modified asphalt.

Figure 7(e) shows the molecular concentration distribution at the asphalt/GO (0 0 1)/SBS compound interface. In the stable state of the asphalt/GO (0 0 1)/SBS compound interface, the upper and lower surfaces of GO (0 0 1) are in contact with the asphalt and the SBS, respectively, and the asphalt hardly contacts the SBS molecules. The interface between GO (0 0 1) and asphalt is similar to the GO (0 0 1)/asphalt interface; saturate, aromatic and resin molecules are sequentially distributed in the transition area between GO (0 0 1) and asphaltene, the most significant binding component to GO(0 0 1) is still the saturate, and the cross-concentration is 3.004 nm. At the interface between GO (0 0 1) and SBS, SBS molecules can bind closely to GO (0 0 1), resulting in a 2.575 nm cross-concentration between them. The binding energy of interface shows that whether GO (0 0 1) is bonded to asphalt or SBS, their interface bonding strengths are higher than that of SBS/asphalt. Therefore, the incorporation of GO improves the binding strength between asphalt and SBS by simultaneously binding with SBS and asphalt, resulting in a more uniform distribution of SBS as well as improving storage stability.

Figure 7(f) shows the molecular concentration distribution at the asphalt/GO (0 1 0)/SBS compound interface. It is different from the asphalt/GO (0 0 1)/SBS compound interface since SBS gradually moves away from GO (0 1 0) during structure relaxation and finally bonds to the asphalt. GO (0 1 0) only binding to the other side of the asphalt and not in contact with SBS, resulting in the formation of a compound interface structure of SBS/asphalt/GO (0 1 0) after stabilization. In this compound interface structure, SBS and GO (0 1 0) are mainly distributed at 7.133 nm and 13.618 nm, respectively, and each component of asphalt fills the 7.133–13.618 nm region to form a transition area. In addition, SBS hardly contacts the asphaltenes, and saturate, aromatic and resin molecules are distributed in sequence between them. The cross-concentration between SBS and asphalt is 3.459 nm. As seen from the structure diagram, some GO sheet layers are exfoliated by the linear saturate and resin molecules, enters the asphalt. Consequently, the downward trend of the S1 and R curves at 11.024–11.457 nm and 11.889–12.321 nm is relatively gentle. The unflaked GO is still in an aggregated state and distributed around the asphalt, but the integrity of the layered structure is reduced. Analysis of the asphalt/GO (0 1 0)/SBS compound interface shows that when both SBS and asphalt are present around the GO (0 1 0), the GO (0 1 0) tends to bond with the asphalt. The SBS molecules are unable to enter the GO layers, GO also cannot peel off in the SBS, and therefore only forms intercalated and exfoliated structures in the asphalt. SBS molecules are poorly protected by GO and some SBS may degrade to small weight molecules during the thermal oxidative ageing^53^.

The volatilization of the saturate and the degradation of the SBS molecules will lead to an increase and a decrease in viscosity, respectively. Although some of the saturate in 70A + 0.2%GO are protected by GO, volatilization will inevitably occur leading to an increase in viscosity of aged 70A + 0.2%GO. For 70A + 5%SBS, the volatilization of saturate is more severe due to the absence of GO and some SBS molecules are also degraded, with the volatilization of saturate more than the degradation of SBS, ultimately leading to an increase in viscosity of aged 70A + 5%SBS. The volatilization of saturate is reduced due to the addition of GO, but the degradation of SBS does not slow down, which may eventually lead to a decrease in viscosity of aged 70A + 5%SBS + 0.2%GO. This is also the reason that incorporation of GO in matrix asphalt improves the anti-ageing performance better than incorporation of GO in SBS-modified asphalt.

**Figure 8:**
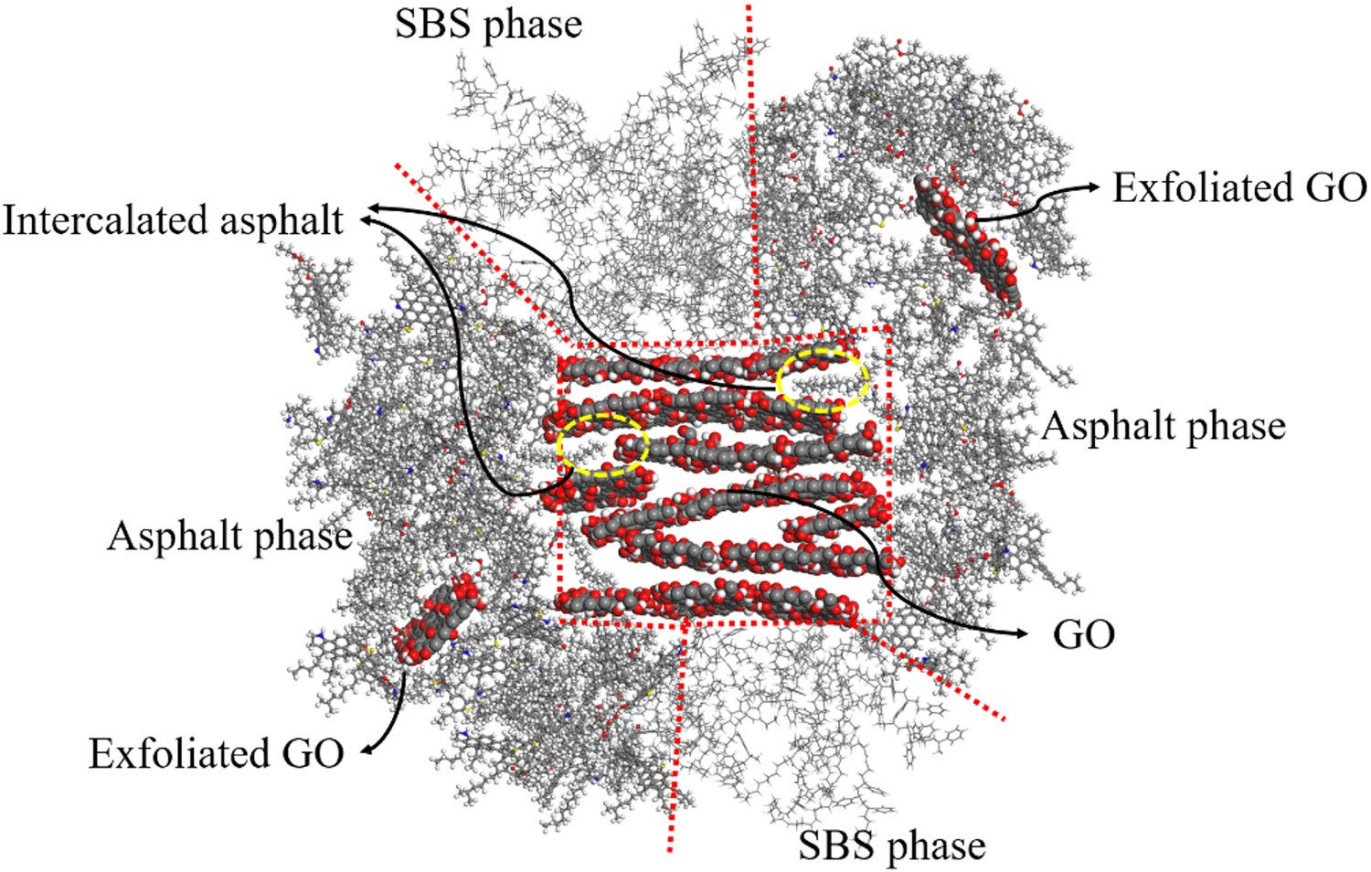
Interface microstructure of GO/SBS compound-modified asphalt.

The interface microstructure of GO/SBS compound-modified asphalt can be obtained based on the results of the interface molecular concentration distribution, as shown in Fig. 8. The asphalt and SBS phases are binding on GO (0 0 1), which improves the bonding strength between SBS and asphalt, resulting in a more uniform distribution of SBS in 70A + 5%SBS + 0.2%GO than in 70A + 5%SBS, and the softening point, low-temperature ductility, viscosity and storage stability properties of 70A + 5%SBS + 0.2%GO are further improved compared to those of 70A + 5%SBS. GO (0 1 0) tends to bind to the asphalt to form intercalated and exfoliated structures, resulting in improved anti-aging performance of asphalt.

#### First-principles calculation of interface

**Figure 9:**
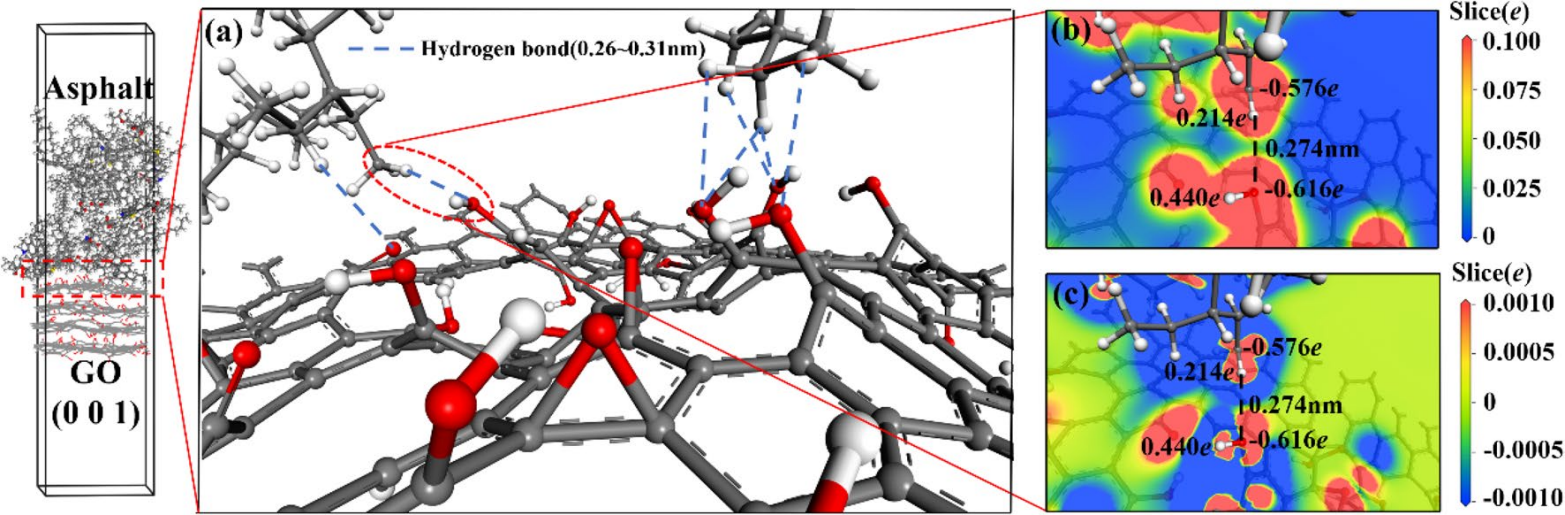
Interface compound mechanism of GO (0 0 1)/asphalt. ((**a**) interface bonding force; (**b**) interface electron density; (**c**) interface electron density difference).

**Figure 10:**
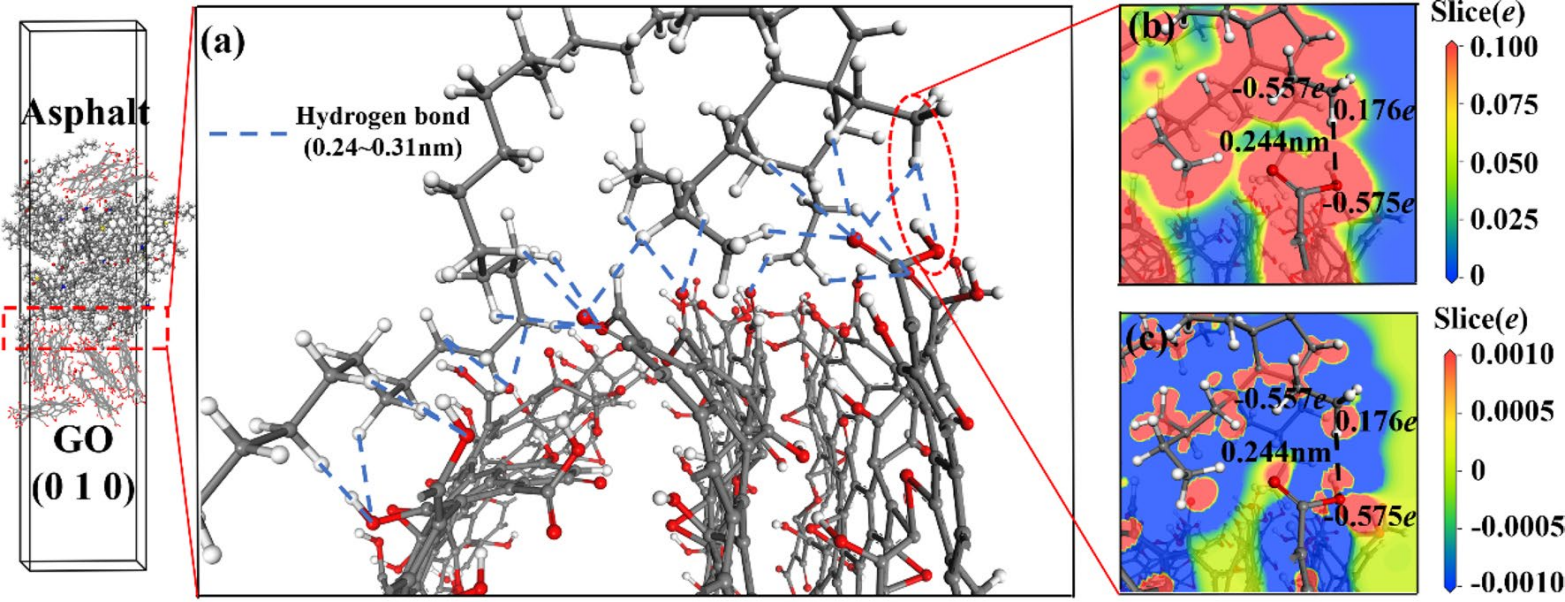
Interface compound mechanism of GO (0 1 0)/asphalt. ((**a**) interface bonding force; (**b**) interface electron density; (**c**) interface electron density difference).

**Figure 11:**
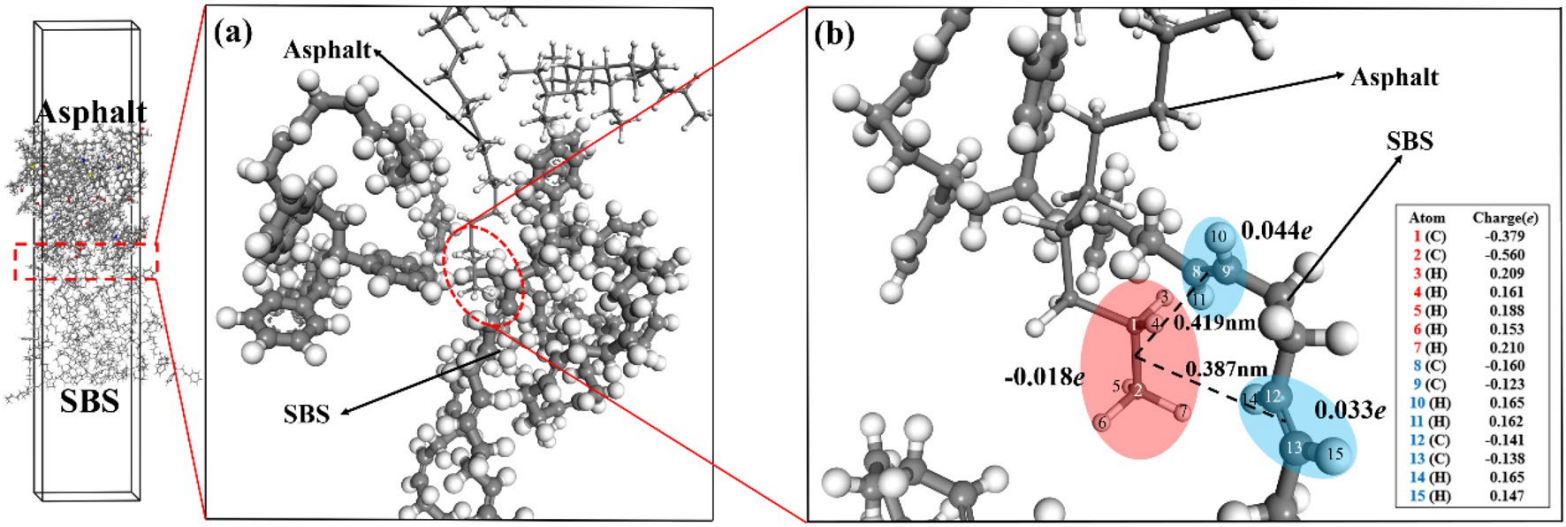
Interface compound mechanism of SBS/asphalt. ((**a**) interface composition; (**b**) interface atomic charge).

**Figure 12:**
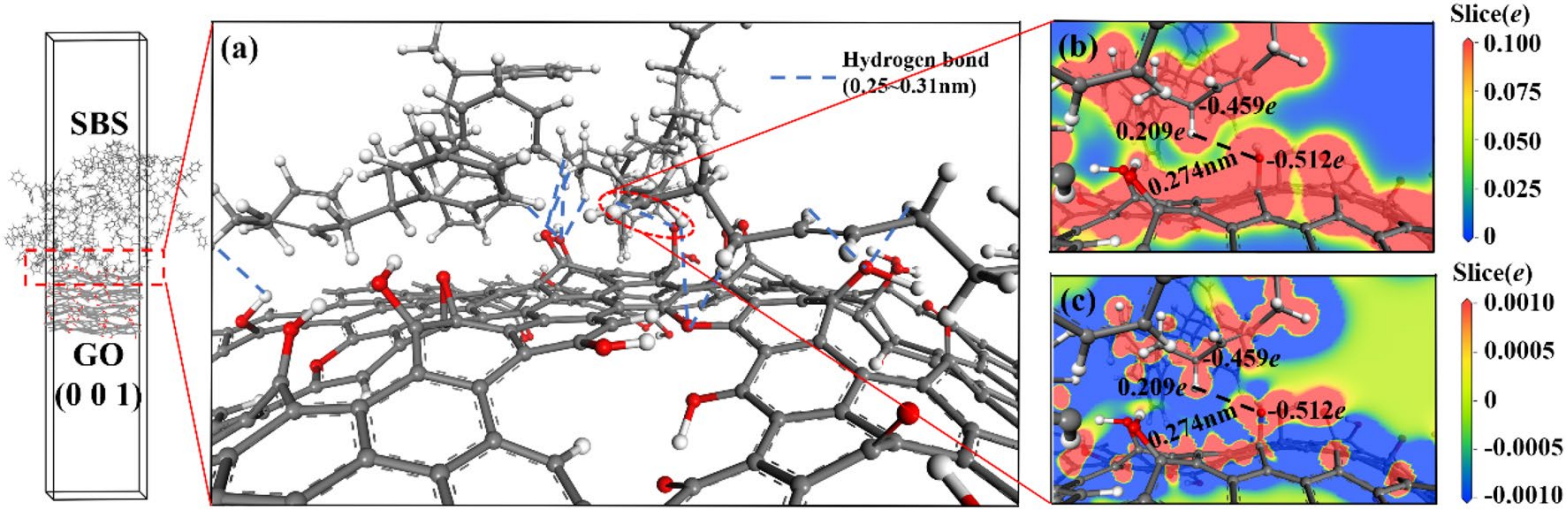
Interface compound mechanism of SBS/GO (0 0 1). ((**a**) interface bonding force; (**b**) interface electron density; (**c**) interface electron density difference).

The local structures of each GO/SBS compound-modified asphalt interface in the steady-state system were extracted and analyzed by first-principles calculations to clarify the physical and chemical properties of the interfaces. Figures 9, 10, 11, 12 show the interface first-principle analysis diagrams of GO (0 0 1)/asphalt, GO (0 1 0)/asphalt, SBS/asphalt and SBS/GO (0 0 1), respectively. The changes in the local structure and energy of the interface during geometry optimization are shown in Fig. S4.

GO has a large number of highly electronegative oxygen atoms with many electrons gathered around it, resulting in an electron-rich state. Moreover, the carbon atoms on the asphalt saturated alkanes or alkane branch chains are more electronegative than the hydrogen atoms that form bonds with the carbon atoms. The outer electron cloud of hydrogen atoms shifts toward the carbon atoms, forming an electron-loss state, as shown in Fig. 9(b,c) and 10(b,c). The carbon atoms on the asphalt alkane or alkane branch chains become hydrogen bond donors, and the oxygen atoms on the GO molecules become hydrogen bond acceptors, forming hydrogen bonds between the GO and asphalt molecules. This hydrogen bond distance ranges from 0.24 nm to 0.31 nm, as shown in Fig. 9(a) and 10(a). GO (0 0 1) mainly distributes hydroxyl and epoxy groups, while GO (0 1 0) mainly distributes carboxyl groups. The oxygen atom concentration of GO (0 1 0) is higher than that of GO (0 0 1), resulting in a higher probability of hydrogen bond formation between GO (0 1 0) and asphalt than that between GO (0 0 1) and asphalt. Moreover, the asphalt linear saturate molecules entering the GO layers from the GO side can simultaneously form hydrogen bonds with both the GO (0 0 1) and the GO (0 1 0), as shown in Fig. 10(a). Therefore, the interface bonding strength between GO (0 1 0) and asphalt is stronger than that between GO (0 0 1) and asphalt.

Figure 11 shows the interface mechanism of SBS/asphalt. Different from GO, SBS molecules mainly binding to asphalt molecules by intermolecular dispersion forces, which are classified as van der Waals forces. Asphalt alkane molecules can diffuse on the surface of SBS and enter the SBS phase, as shown in Fig. 11(a). The end of the asphalt alkane molecule has a negative charge of 0.018e, and the adjacent carbon–carbon double bond fragments of the two butadiene fragments on the SBS molecule have a positive charge of 0.044e and 0.033e, respectively, as shown in Fig. 11(b). This local difference in positive and negative charges results in an intermolecular dispersion force between the asphalt alkane molecules and the SBS molecules. Figure 12 shows the interface mechanism of SBS/GO (0 0 1). Similar to the interface between GO and asphalt, the hydrogen bonds between SBS and GO (0 0 1) are also dominant, as shown in Fig. 12(a). The hydrogen atoms bonded to the saturated carbon atoms on the SBS butadiene fragment are also in an electron-loss state, meaning that the saturated carbon atoms on the SBS molecules become hydrogen bond donors, the oxygen atoms on GO (0 0 1) still serve as hydrogen bond acceptors, and hydrogen bond interactions are formed between the SBS molecules and GO (0 0 1), as shown in Fig. 12(b,c).

In summary, the GO (0 0 1)/asphalt, GO (0 1 0)/asphalt and GO (0 0 1)/SBS interfaces are mainly binding by hydrogen bonds, especially the GO (0 1 0)/asphalt interface. The asphalt linear saturate molecules that enter the GO layers can simultaneously form hydrogen bonds with GO (0 0 1) and GO (0 1 0), while the SBS/asphalt interface is mainly binding by van der Waals forces. The hydrogen bond strength is higher than that of van der Waals forces, resulting in a higher binding strength between GO and asphalt or SBS than that between SBS and asphalt. All the interfaces in the GO/SBS compound-modified asphalt do not exhibit chemical reactions, which is consistent with the FTIR analysis results. Since SBS is mainly binding to the saturate component of asphalt and generates van der Waals forces, in addition, the hydrogen bond donors are provided mainly by the asphalt alkanes or the carbon atoms on the alkane branches. As a result, both SBS and GO can bring a certain degree of adsorption to the alkanes or alkane linear chains, thus reducing the short-range order of these chains in asphalt. This result supports the fact that the γ peak of 70A is stronger than those of 70A + 0.2%GO, 70A + 5%SBS and 70A + 5%SBS + 0.2%GO in the XRD characterization.

#### Glass transition temperature

**Figure 13:**
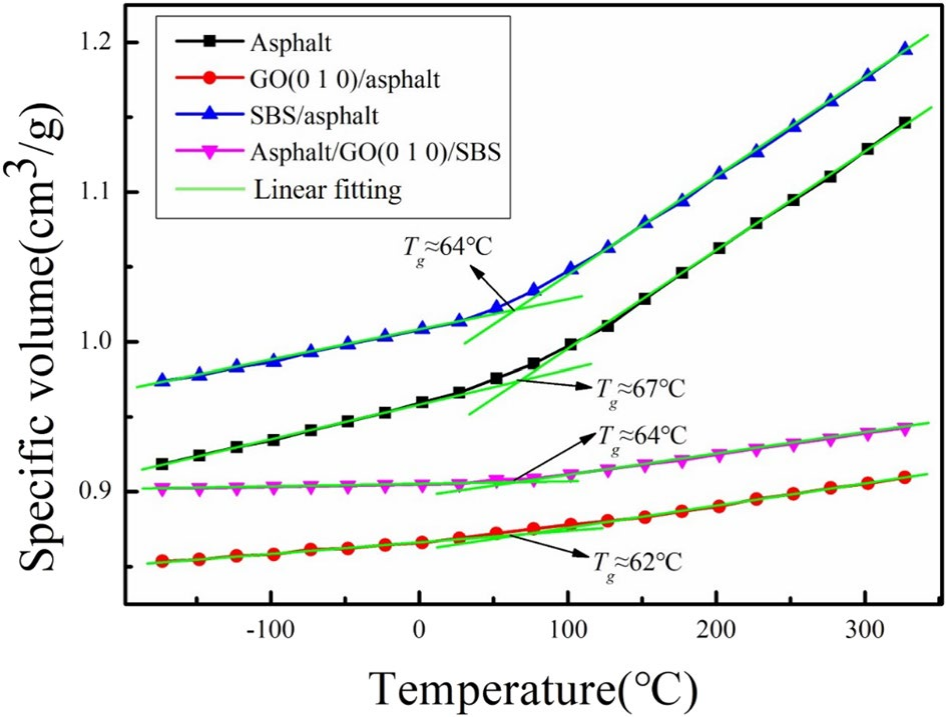
Specific volume-temperature data and glass transition temperatures for asphalt, GO (0 1 0)/asphalt, SBS/asphalt and asphalt/GO (0 1 0)/SBS.

Temperature at which mechanical states of asphalt change from viscoelastic state to glass state is called the glass transition temperature (*T*_*g*_), and *T*_*g*_ is a significant parameter that determines the viscoelastic properties of asphalt^54^. Considering that the binding strength between GO (0 1 0) and asphalt is higher than that between GO (0 0 1) and asphalt, the glass transition temperatures of the asphalt, GO (0 1 0)/asphalt, SBS/asphalt and asphalt/GO (0 1 0)/SBS block models (not given vacuum layer) are analyzed by simulation of the specific volume-temperature relationship. Simulation was performed using NPT ensemble, with temperatures ranging from − 173 °C to 327 °C, and temperature intervals of 25 °C. The *T*_*g*_ was determined by finding the point of intersection of the fitted lines in the rubbery and glassy regions on this specific volume-temperature plot^38^. As shown in Fig. 13, the *T*_*g*_ of the asphalt model is about 67 °C. The results stay consistent with the range of the reference (25 °C–80 °C) from Zhang and Greenfield^49^. The* T*_*g*_ of GO (0 1 0)/asphalt, SBS/asphalt and asphalt/GO (0 1 0)/SBS models are about 62 °C, 64 °C and 64 °C, respectively. *T*_*g*_ is essentially unchanged when GO or SBS is incorporated into the asphalt, indicating that both GO and SBS do not significantly influence the transition from the glass state to the rubbery state of the asphalt, and the synergistic modification of asphalt by GO/SBS is not caused by changes in *T*_*g*_.

## Conclusion

1. GO/SBS modification effect on asphalt is significantly superior to that of GO or SBS individually. GO/SBS provides a synergistic modification effect, improving the softening point, low-temperature ductility, viscosity, anti-ageing and storage stability of the compound modified asphalt. The optimum contents of GO and SBS are 0.2wt% and 5wt%, respectively.
2. The interface binding strength between SBS and asphalt is the weakest, followed by that between GO (0 0 1) and asphalt or SBS, and the strongest interface bonding strength appears between GO (0 1 0) and asphalt or SBS. The incorporation of GO can effectively enhance the bonding strength between SBS and asphalt by GO simultaneously binding with asphalt and SBS, promoting a more uniform distribution of SBS in the asphalt, resulting in a synergistic modification effect of GO/SBS.
3. The linear alkanes of the asphalt enter the GO layer to form an intercalated structure, and some GO sheet layers enter the asphalt to form an exfoliated structure. These two structures prevent the ageing of asphalt, but they are only formed between asphalt and GO, therefore, the ageing of SBS cannot be improved, resulting in incorporation of GO in matrix asphalt improves the anti-ageing performance better than incorporation of GO in SBS modified asphalt.
4. The GO (0 0 1)/asphalt, GO (0 1 0)/asphalt and GO (0 0 1)/SBS interfaces are mainly binding by hydrogen bonds, while the SBS/asphalt interface is mainly binding by van der Waals forces. Therefore, the interface bonding strength between SBS and asphalt is the weakest. All interfaces do not exhibit chemical reactions, meaning that physical modifications dominate the modification effect of GO/SBS on asphalt.

## Supplementary Information

Supplementary Information.

## Data Availability

The data that supports the findings of this study are available from the corresponding author upon reasonable request.
