# Supplementary material for: Research on the synergistic modification effect and the interface mechanism of GO/SBS compound-modified asphalt based on experiments and molecular simulations
Source: Sci Rep. 2023 Mar 1;13:3496. doi: 10.1038/s41598-023-30593-0 (PMC9977838; doi:10.1038/s41598-023-30593-0)
Supplement: Supplementary file 1 — Supplementary Information. [file 41598_2023_30593_MOESM1_ESM.docx]

**Supplementary materials**

**Raw materials**

The 70A matrix asphalt used in this study was produced by Hunan Baoli Asphalt Co., Ltd. (Hunan China), and the physical properties of this asphalt are listed in **Table S1**. The 791-H linear SBS produced by Sinopec Baling branch company was used as a polymer modifier, and the ratio of styrene to butadiene was 30:70. GO with fewer than 5 layers was provided by the Chinese Academy of Sciences Chengdu Organic Chemistry Co., Ltd. (Chengdu, China). The purity of GO was higher than 97 wt%, and the thickness and lamellar diameter of GO were approximately 0.55 nm-2.0 nm and 3μm-10μm, respectively.

**Table S1**. Physical properties of 70A matrix asphalt.

| Technical Parameter | Units | Test Result | Method |
| --- | --- | --- | --- |
| Softening point | ℃ | 48.6 | ASTM D36 |
| Penetration (25°C) | 0.1 mm | 71.0 | ASTM D5 |
| Ductility (25°C) | cm | >100 | ASTM D113 |
| Ductility (5°C) | cm | 8.5 | ASTM D113 |
| Viscosity (135°C) | mPs·s | 359.4 | ASTM D4402 |

**Characterization**

The structures of the GO and the modified asphalts were analyzed using XRD performed on a Philips PW 1710 diffractometer with Cu Kα1 radiation. The diffractograms were scanned from 5° to 40° in the 2*θ* range of 0.01°. Characterization of the infrared spectra of unmodified and modified asphalts was conducted using an FTIR spectrometer (Nicoet 460), and the wavenumber ranged from 400 cm^-1^ to 4000 cm^-1^ with a resolution of 2 cm^-1^. The fluorescent micrographs of the modified asphalt were characterized using a fluorescent microscope with equipment model Zeiss Vert. A1, and the magnification of the micrographs was 200x.

**Asphalt properties tests**

The physical properties of asphalts, including the softening point, penetration (at 25°C), ductility (at 5°C and 25°C) and separation, were tested according to the ASTM D36, ASTM D5, ASTM D113 and ASTM D5976 methods, respectively. The softening point test was performed on the SYD-2806H automatic asphalt softening point tester, the penetration test was performed on the SYD-2801E1 penetration tester at 25°C and the ductility test was performed on the SYD-4508C asphalt ductility tester at 5°C or 25°C. According to the ASTM D2872 standard, a rotation thin film oven test (RTFO) was conducted to simulate asphalt aging. The RTFO was performed on equipment model SYD-0610, the aging temperature, aging time and air flow rate were 163°C, 75 minutes and 4000 mL/min, respectively. An Anton Paar 300-H Brookfield viscometer was used to measure the rotational viscosity of asphalt in the temperature range 70℃-150℃ according to ASTM D 4402. All modified asphalt was prepared in three equal samples for different asphalt properties tests.


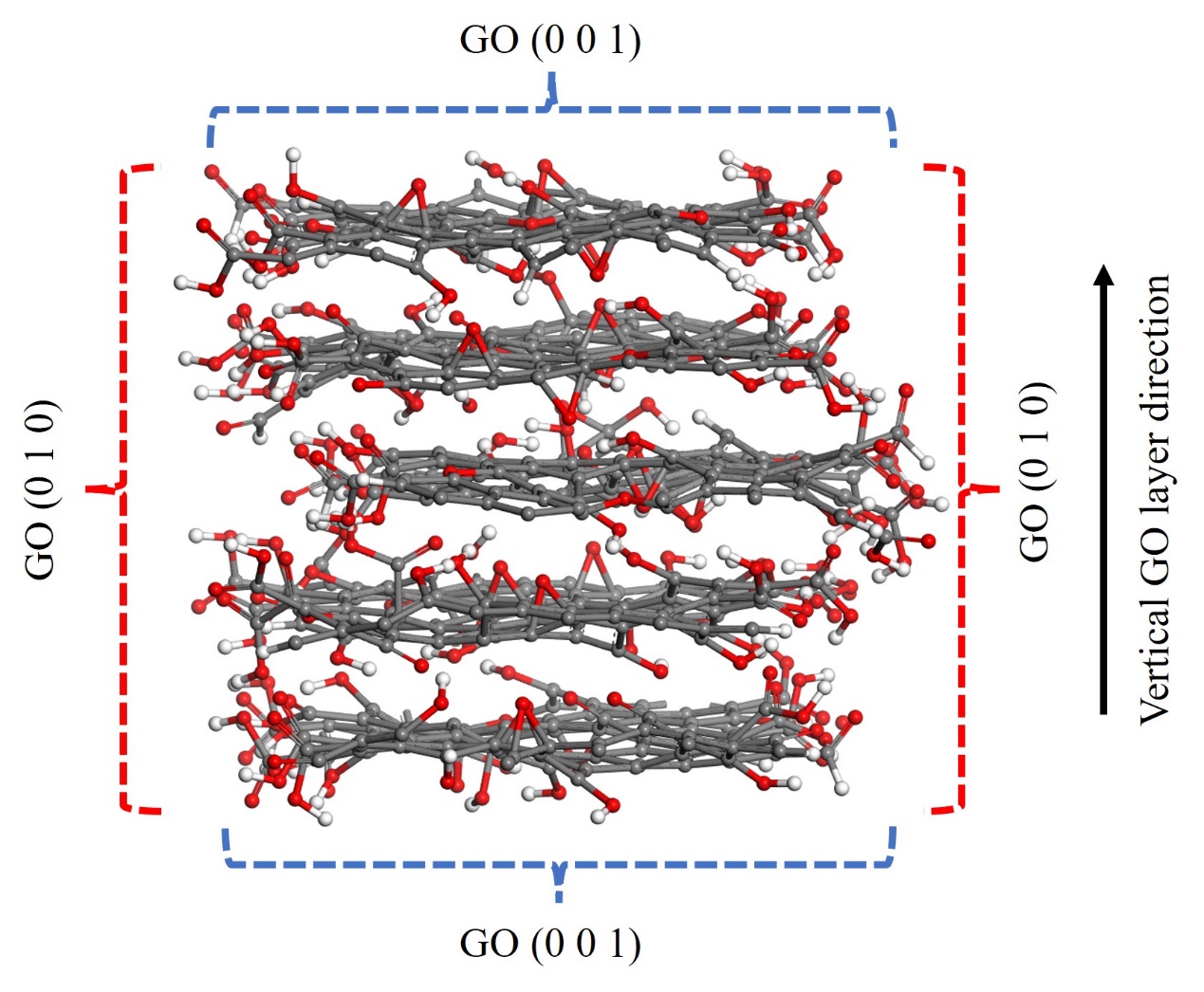


**Figure S1**. Surface of GO(0 0 1) and GO(0 1 0) in molecular simulations.

**Molecular dynamics simulation**

**
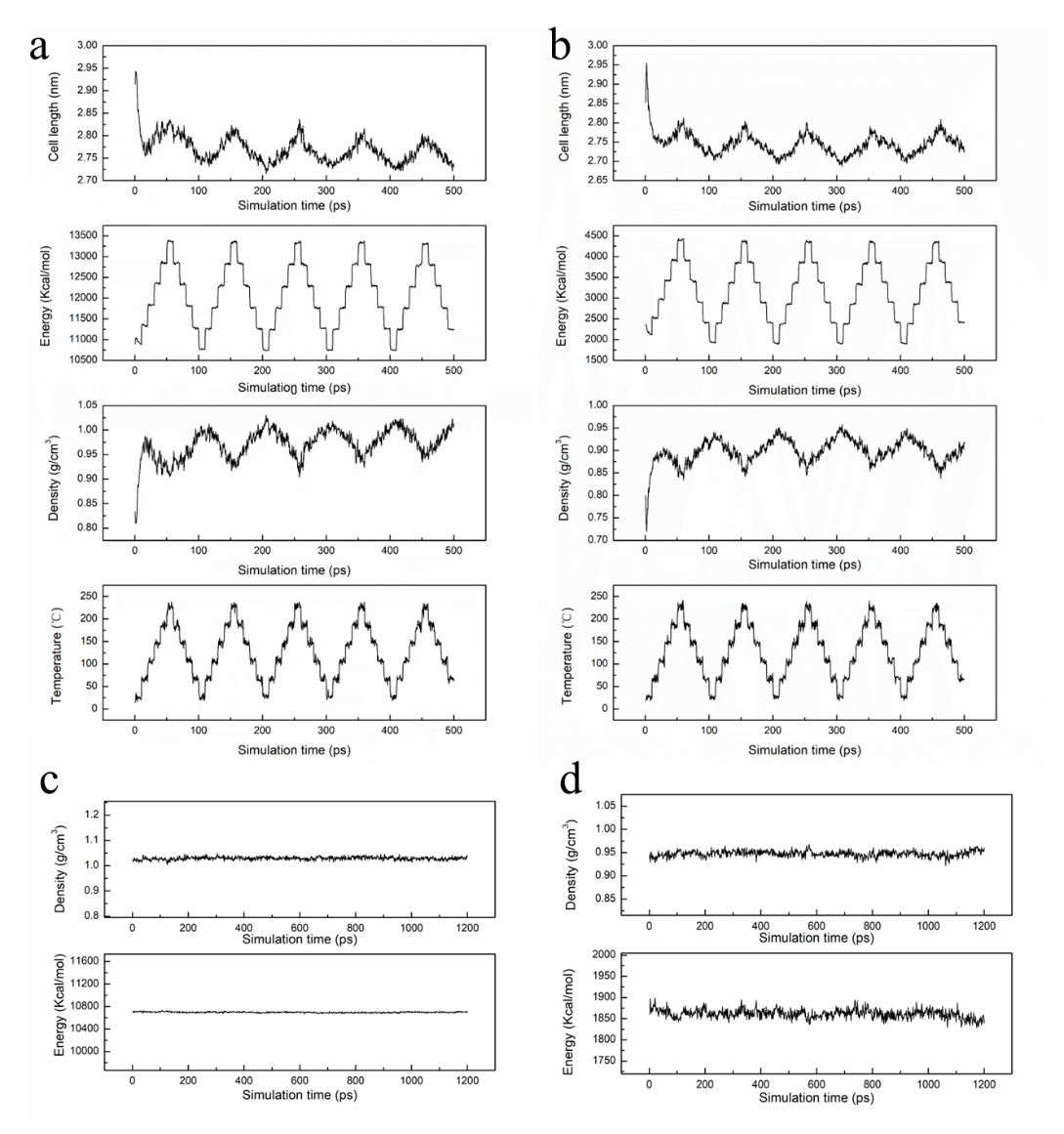
**

**Figure S2**. Changes in system properties during annealing of asphalt (a) and SBS (b) at 27-227°C; changes in system properties during molecular dynamics relaxation of asphalt (c) and SBS (d) at 25°C.

There are no significant fluctuations in the energy and density of the system during the molecular dynamic relaxation of the asphalt and SBS in **Figure S2 (c) (d)**. This is probably due to the fact that the asphalt and SBS have been annealed at 27℃-227°C for 5 cycles prior to the molecular dynamic relaxation, and the annealing end temperature (27°C) is very close to the molecular dynamic relaxation temperature (25°C). Annealing is a process in which sufficient energy is input into the system through high temperatures to eliminate unreasonable structures and is also the process of structural relaxation. As a result, the asphalt and SBS are able to approach equilibrium at the beginning of the molecular dynamics relaxation and without significant fluctuations in the energy and density of the system.

**
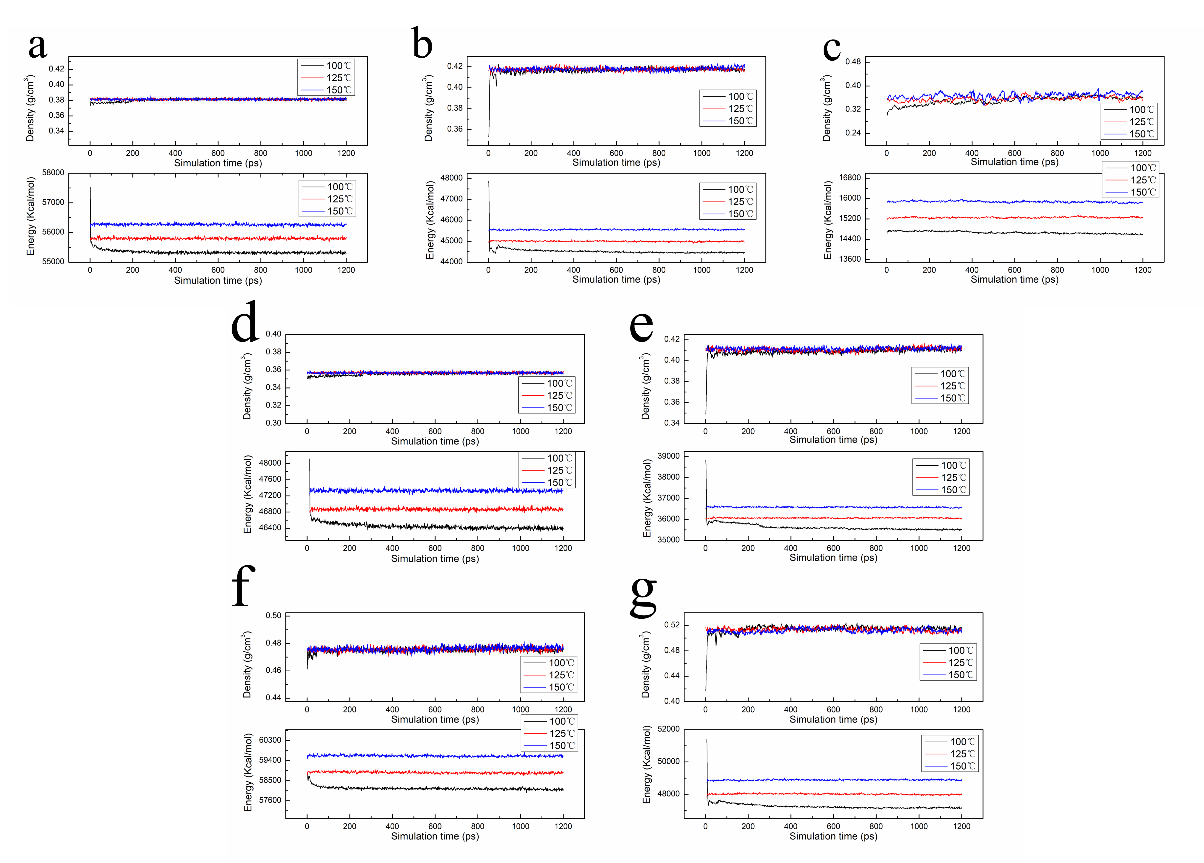
**

**Figure S3**. Changes in system properties of GO (0 0 1)/asphalt (a), GO (0 1 0)/asphalt (b), SBS/asphalt (c), GO (0 0 1)/SBS (d), GO (0 1 0)/SBS (e), asphalt/GO (0 0 1)/SBS (f) and asphalt/GO (0 1 0)/SBS (g) during molecular dynamics relaxation at 100°C, 125°C and 150°C respectively.

**DFT-D**

Non-covalent forces such as hydrogen bonding and van der Waals (vdW) interactions are long-range interactions, the correct long-range interaction tail is absent from all popular gradient corrected exchange-correlation functionals of Density functional theory (DFT). In order to study hydrogen bonding and van der Waals interactions in the system, it is necessary to add corresponding correction terms. DFT-D is a density functional that introduces a long-range interaction correction term, which is called dispersion correction.

The physical and chemical effects at the interface need to be investigated in this study, so the DFT method is used. In addition, non-covalent forces are ubiquitous in asphalt systems and are critical to the stability of asphalt materials, so DFT-D is used for dispersion correction.

**Table S2**. Binding energies between each interface of the GO/SBS compound-modified asphalt.

| Temperature(℃) | 100 | 125 | 150 |
| --- | --- | --- | --- |
| GO(0 0 1)/Asphalt  (Kcal/mol) | -160.99 | -163.93 | -164.66 |
| GO(0 1 0)/Asphalt  (Kcal/mol) | -319.34 | -324.58 | -332.11 |
| SBS/Asphalt  (Kcal/mol) | -113.43 | -114.08 | -82.72 |
| GO(0 0 1)/SBS  (Kcal/mol) | -184.68 | -171.08 | -180.22 |
| GO(0 1 0)/SBS  (Kcal/mol) | -375.30 | -376.28 | -372.98 |
| Ashphalt-GO(0 0 1)/SBS  (Kcal/mol) | -161.63 | -160.80 | -149.64 |
| Ashphalt/SBS-GO(0 0 1)  (Kcal/mol) | -160.88 | -156.14 | -155.18 |
| Ashphalt-GO(0 1 0)/SBS  (Kcal/mol) | -135.01 | -141.75 | -130.38 |
| Ashphalt/SBS-GO(0 1 0)  (Kcal/mol) | -344.60 | -354.19 | -331.25 |


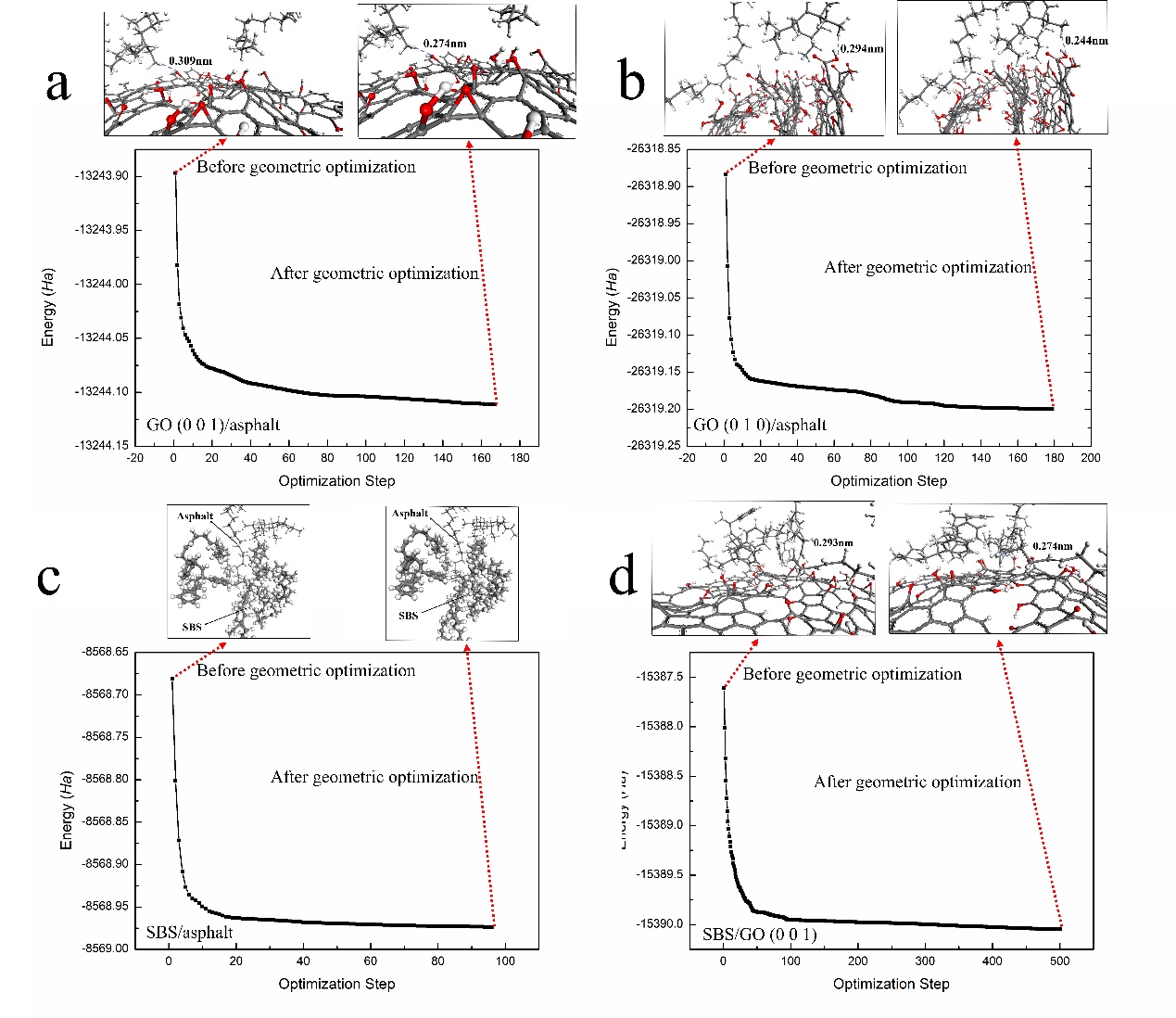


**Figure S4**. Structure and energy changes in the local structure of interfaces during geometry optimization.
